# *Galleria mellonella* as an Infection Model for *Bacillus anthracis* Sterne

**DOI:** 10.3389/fcimb.2019.00360

**Published:** 2019-10-18

**Authors:** Jacob A. Malmquist, Madison R. Rogan, Shauna M. McGillivray

**Affiliations:** Department of Biology, Texas Christian University, Fort Worth, TX, United States

**Keywords:** *Galleria mellonella*, *Bacillus anthracis*, Sterne, infection model, hydrogen peroxide, virulence

## Abstract

Understanding bacterial virulence provides insight into the molecular basis behind infection and could identify new drug targets. However, assessing potential virulence determinants relies on testing in an animal model. The mouse is a well-validated model but it is constrained by the ethical and logistical challenges of using vertebrate animals. Recently the larva of the greater wax moth *Galleria mellonella* has been explored as a possible infection model for a number of pathogens. In this study, we developed *G. mellonella* as an infection model for *Bacillus anthracis* Sterne. We first validated two different infection assays, a survival assay and a competition assay, using mutants containing disruptions in known *B. anthracis* virulence genes. We next tested the utility of *G. mellonella* to assess the virulence of transposon mutants with unknown mutations that had increased susceptibility to hydrogen peroxide in *in vitro* assays. One of these transposon mutants also displayed significantly decreased virulence in *G. mellonella*. Further investigation revealed that this mutant had a disruption in the petrobactin biosynthesis operon (*asbABCDEF*), which has been previously implicated in both virulence and defense against oxidative stress. We conclude that *G. mellonella* can detect attenuated virulence of *B. anthracis* Sterne in a manner consistent with that of mammalian infection models. Therefore, *G. mellonella* could serve as a useful alternative to vertebrate testing, especially for early assessments of potential virulence genes when use of a mammalian model may not be ethical or practical.

## Introduction

*Bacillus anthracis* is a gram-positive, spore-forming bacterium that causes the deadly disease anthrax. Major virulence determinants include the lethal and edema toxins encoded on the pXO1 plasmid and the capsule encoded on the pXO2 plasmid. Additionally, there are putative virulence genes encoded within the chromosome (Read et al., 2003) and a number of groups have experimentally demonstrated that chromosomally encoded genes are essential for virulence (Gat et al., 2005; McGillivray et al., 2009; Kern and Schneewind, 2010; Chitlaru et al., 2011; Jenkins et al., 2011). The Sterne strain of *B. anthracis* lacks the pXO2 plasmid (pXO1^+^, pXO2^−^) and is avirulent in humans and normal mice due to increased sensitivity to complement, but it is a well-established alternative to the fully virulent *B. anthracis* (pXO1^+^, pXO2^+^) strains which can only be studied in a limited number of labs (Harvill et al., 2005). Complement-deficient mice (A/J, DBA/J) are commonly used as the model system for the Sterne strain (Welkos and Friedlander, 1988), but there are ethical and logistical constraints to using any vertebrate model. This is particularly true in preliminary studies for which it is difficult to justify the use of large numbers of vertebrate animals. While *in vitro* and cell-based assays have their place, an animal model is eventually needed for *in vivo* infection studies.

Invertebrate models have shown promise as infection models. They are inexpensive and easy to maintain, and they possess conserved innate immune system components (Glavis-Bloom et al., 2012). *C. elegans* has previously been used with *B. anthracis* Sterne as well as other bacterial pathogens (Kho et al., 2011; Glavis-Bloom et al., 2012; Franks et al., 2014), but there are limitations to this model. *C. elegans* cannot be incubated at 37°C, the optimal temperature for bacterial pathogens, and the route of infection is via ingestion which is convenient, but can lead to uneven exposure to pathogen. It also does not seem to possess phagocytic cells analogous to macrophages or neutrophils (Gravato-Nobre and Hodgkin, 2005). Moreover, infection of *C. elegans* by *B. anthracis* Sterne also requires additional stresses to be placed on the worm including exposure to the Cry5B toxin and incubation at abnormally high temperatures for the worm (Kho et al., 2011; Franks et al., 2014). The larva of the greater wax moth *Galleria mellonella* has recently emerged as a desirable infection model with several advantageous physical and molecular features. These include a size sufficient for direct injection; the ability to incubate the larvae at 37°C; and the presence of innate immune defenses that are analogous to mammalian defenses including phagocytic hemocytes, the production of reactive oxygen species (ROS), opsonizing proteins that recognize conserved microbial patterns, and the production of antimicrobial peptides (AMPs) such as lysozyme (Kavanagh and Reeves, 2004; Tsai et al., 2016). *G. mellonella* has been used as an infection model for both gram-positive and gram-negative bacteria including *Staphylococcus aureus, Streptococcus pyogenes, Enterococcus faecalis, Pseudomonas aeruginosa, Escherichia coli*, and *Acinetobacter baumannii* to name a few. *In vivo* studies utilizing *G. mellonella* have been used to both identify new virulence factors and evaluate potential antibacterial compounds (Tsai et al., 2016).

In this study, we developed *G. mellonella* survival and competition assays for use with *B. anthracis* Sterne and validated these infection models using mutants with disruptions in previously identified virulence genes of *B. anthracis*. We then conducted a small proof-of-concept screen with transposon mutants to demonstrate the utility of *G. mellonella* for studying potential virulence determinants of *B. anthracis* Sterne. We find that *G. mellonella* is a suitable animal model for *B. anthracis* Sterne and that this could be a practical and ethical manner in which to assess virulence.

## Methods

### Bacterial Growth Conditions and Strains

*Bacillus anthracis* Sterne strain 34F2 (pX01^+^, pX02^−^) was cultured in Brain-Heart Infusion (BHI) media (Hardy Diagnostics, Santa Maria CA, USA) at 37°C under aerobic conditions. Antibiotics were used at the following concentrations: *E. coli*: erythromycin 500 μg/ml (Erm500); *B. anthracis*: erythromycin 5 μg/ml (Erm5) and kanamycin 50 μg/ml (Kan50) (all Sigma, St. Louis MO, USA). Construction of *B. anthracis* strains ΔpXO1, Δ*clpX*, Δ*yceGH* and the transposon library were previously described (van Sorge et al., 2008; McGillivray et al., 2009; Franks et al., 2014). The remaining strains were constructed in this study.

### Construction of Δ*purH*, Δ*mntA*, and Δ*htrA* Virulence Mutants

To disrupt the *purH, mntA*, and *htrA* genes by insertional mutagenesis, an internal fragment of each gene was amplified via PCR using primers engineered with 5′ extensions containing restriction sites (described in Table 1). Each amplicon was digested and ligated into the temperature-sensitive plasmid pHY304 (all enzymes NEB, Ipswich, MA, USA), transformed into the *E. coli* strain MC1061 (Lucigen, Middleton, WI, USA), and plated under Erm500 selection at 30°C. Plasmids were then extracted via miniprep (IBI Scientific, Peosta IA, USA), transformed into methylation-deficient *E. coli* strain GM2163, and plated on Erm500 selection. GM2163 grown plasmids were extracted, transformed into electrocompetent *B. anthracis* Sterne as previously described (Koehler et al., 1994), and plated under Erm5 selection at 30°C. *B. anthracis* Sterne containing the insertional mutant plasmid were passed two times at 37°C in BHI-Erm5 to force plasmid integration. Integration into the bacterial chromosome was confirmed by PCR using the pHY304-Fwd primer and the gene-specific confirmation primer located downstream of the original amplicon (Table 1).

**Table 1:** Primers and oligonucleotides used in this study.

**Name**	**Sequence**
purH Fwd-XhoI	5′-ACAGTCTCGAGAAAGAGAACGGTGAAGTAGCAGAG-3′
purH Rev-HindIII	5′-GACTAAGCTTCATGAATATCCGTACCTACTCCAACA-3′
mntA Fwd-XhoI	5′-ACAGTCTCGAGAACCCGCATGAATATGATCCACTAC-3′
mntA Rev-HindIII	5′-GACTAAGCTTCGTTTCTCCTCAGGGATTTGATG-3′
htrA Fwd-EcoRI	5′-ACGTCTCGAGTGCAATGCAACCGACAGG-3′
htrA Rev-XhoI	5′-tacgGAATTCGCACGCTTATCGCCATCGA-3′
pHY304 Fwd	5′-ACGACTCACTATAGGGCGAATTGG-3′
ΔpurH confirm Rev	5′-GCTTGCGCTTGTCGCTTT-3′
ΔmntA confirm Rev	5′-AGGTAATGGGATTTGGGAAGGTG-3′
ΔhtrA confirm Rev	5′-AACGACACCTACTCCAAGAGC-3′
Linker 1	5′-TTTCTGCTCGAATTCAAGCTTCTAACGATGTAC GGGGACACATG
Linker 2	5′-TGTCCCCGTACATCGTTAGAACTACTCGTACCA TCCACAT-3′
Himar 1-2 Fwd	5′-GGGAATCATTTGAAGGTTGGTACT-3′
Y-linker Rev	5′-CTGCTCGAATTCAAGCTTCT-3′
Himar 1-4 Fwd	5′-TATGCATTTAATACTAGCGAC-3′
4D5 Tn confirm Rev	5′-TCGCCTCTTGCACACCTTCC-3′

### Validation of *in vitro* Phenotypes of Δ*purH*, Δ*mntA*, and Δ*htrA*

**Δ*purH*:** Overnight parental and Δ*purH* cultures were washed and resuspended in PBS then diluted in minimal R-medium (Ristroph and Ivins, 1983) and BHI at a 1:15 dilution. Cultures were incubated at 37°C under shaking conditions. The optical density (OD) at wavelength 600 nm was determined after 24 h to quantify bacterial growth. **Δ*mntA***: Parental and Δ*mntA* cultures were grown overnight and then diluted to final concentration of 1:50 in BHI containing 0 or 0.005% H_2_O_2_ in 96-well plates. Plates were incubated at 37°C under static conditions for 24 h and growth assessed by measuring OD. **Δ*htrA*** ***H***_**2**_***O***_**2**_: Parental and Δ*htrA* cultures were grown to log phase (OD = 0.4) and then diluted 1:20 in BHI containing 0 or 0.0025% H_2_O_2_ in 96-well plates. Plates were incubated at 37°C under static conditions and growth was assessed at 8 h by measuring OD. **Δ*htrA temperature*:** Parental and Δ*htrA* cultures were grown to log phase (OD = 0.4) and then diluted 1:100 in BHI and grown under shaking conditions at 37°C or 44°C. Growth was monitored and OD measured every hour for 6 h.

### *G. mellonella* Infection Assays

*Galleria mellonella* larvae were obtained from Rainbow Mealworms (www.rainbowmealworms.net), stored at 4°C to induce torpidity, and used within 5 days of receipt. Only larvae weighing 190–220 mg were used in assays. Injections were done through the posterior cuticle using an automated pump (New Era Pump Systems NE-500, Farmingdale, NY, USA) and a 27-gauge needle.

#### Survival Assay

**Figure 1:**
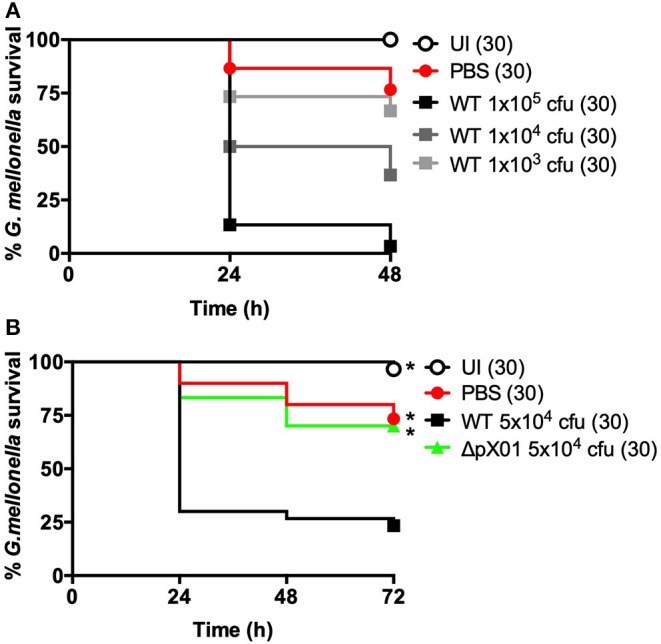
*B. anthracis* Sterne can infect *G. mellonella* larvae. Larvae were left uninjected (UI), injected with PBS, or injected with *B. anthracis* Sterne using approximately the number of colony forming units (cfu) indicated on the graphs. Each infection was repeated three independent times and total number of larvae for each condition are indicated in parentheses. **(A)** Survival comparison using different doses of wild-type (WT). **(B)** Virulence comparison of wild-type (WT) and *B. anthracis* lacking the pXO1 plasmid (ΔpXO1). ^*^Indicates statistically different survival than wild-type, *p* < 0.0167 by the Mantel-Cox log rank test.

*Bacillus anthracis* Sterne strains were grown to an OD 0.4 in BHI (~1 × 10^7^ colony forming units [cfu] per ml), washed and resuspended in PBS, and then diluted prior to injection. For the dose curve in Figure 1A, bacteria were diluted in PBS 1:100, 1:10, or used undiluted; for all other survival assays, bacteria were diluted 1:2 in PBS. Ten microliters of diluted bacteria were injected per larva and 10 larvae were injected for each condition. The starting inoculum was confirmed through serial dilution and enumeration of cfu. After injection, larvae were observed at room temperature for 15–30 min to ensure they recovered from injection and were transferred to an incubator at 37°C. Surviving larvae were counted at 24, 48, and 72-h post-injection.

#### Competition Assay

*Bacillus anthracis* Sterne strains were grown to log phase (OD = 0.4) and washed and resuspended in PBS. Mutant strains were mixed with the parental (wild-type) strain at a 1:1 ratio. Prior to injection, the mixed culture was plated on both plain and antibiotic-containing media (Erm5 for Δ*htrA*; Kan50 for Δ*yceGH*) to ensure an equal starting ratio. Ten microliters of the mixed culture (~1 × 10^5^ total cfu) was injected into each larva and 5 larvae were injected for each condition. After injection, larvae were observed for 15–30 min at room temperature to ensure they recovered from injection and then were incubated at 37°C for 6 h. After incubation, larvae were briefly rinsed in 70% ethanol followed by sterile water and transferred to 2 ml screw-top tubes containing 400 μl PBS and 1 mm ceramic beads (Fox Industries, Fairfield NJ, USA). Larvae were homogenized using the Fast Prep-24 (MP Biomedicals, Solon, OH, USA) with two pulses at 4.5 m/s for 45 s. Surviving bacteria were enumerated by serial dilution plating on BHI (total cfu) and BHI-Erm or BHI-Kan media (mutant cfu). Wild-type cfu were determined by subtracting the antibiotic-resistant mutant cfu from the total cfu and the percent recovery was calculated by dividing the number of wild-type or mutant cfu by the total number of cfu and multiplying by 100.

### H_2_O_2_ Assays Using Transposon Mutants

Transposon mutants were grown overnight in 96-well plates and then diluted 1:100 into a new 96-well plate containing 200 μl of 0 or 0.01% H_2_O_2_ in BHI. Plates were incubated overnight at 37°C under static conditions and growth was measured at OD 0.4.

### Identification of Site of Transposon Insertion

The site of transposon insertion was identified using the Y-linker method (Kwon and Ricke, 2000). Briefly, 18 μl of the linker 2 oligonucleotide (100 μM) was phosphorylated with PNK (2 μl enzyme, 4 μl 10x T4 ligase buffer, 16 μl H_2_O) at 37°C for 1 h and then denatured at 65°C for 20 min. To form the Y-linker, 18 μl of linker 1 oligonucleotide (100 μM) was then added to the mixture and it was incubated at 95°C for 5 min before being slowly cooled to room temperature to allow for linker 1 and 2 to anneal. Genomic DNA was extracted using phenol/chloroform and 5 μg was digested with *NlaIII*. Two-hundred nanograms of digested DNA was ligated to 5 μl of the annealed Y-linker using 1 μl T4 ligase in a 20 μl total reaction (all enzymes NEB, Ipswich, MA, USA). The ligation was diluted to a final volume of 200 μl and then heat denatured, and 2 μl was used for template in a PCR reaction using the Y-linker primer and the transposon specific primer Himar 1–2. PCR products were purified and sent for sequencing using the Himar 1–4 primer. Results were BLASTed against the *B. anthracis* genome and the site of insertion was confirmed using the Himar 1–2 primer and a primer located downstream of the insertion site. All primers/oligonucleotides are listed in Table 1.

### Statistics

GraphPad Prism (San Diego, CA, USA) was used for all statistical analysis. To correct for error caused by multiple comparisons in the survival curves, the level of statistical significance was set as 0.05 divided by the number of comparisons being made.

## Results

### Survival Assay Using *G. mellonella*

Our first question was whether *B. anthracis* Sterne could reliably infect *G. mellonella*. To test this, we adapted a previously described protocol (Ramarao et al., 2012) using *G. mellonella* ordered from an online bait store (Rainbow Mealworms). We selected *G. mellonella* larvae weighing between 190 and 220 mg in order to minimize differences due to weight and standardize larval age [last instar larvae are between 180 and 250 mg (Ramarao et al., 2012)]. Before injecting larvae with the pathogen, we stored them at 4°C overnight to induce torpidity, which renders them immobile and makes them easier to inject. We injected 10 μl of varying doses of *B. anthracis* into the posterior cuticle of each larva and then allowed them to recover for 15–30 min at room temperature before placing them at 37°C and monitoring larval survival every 24 h. Larval death is easy to assess as they turn black shortly before death due to melanization (Tsai et al., 2016). Under these conditions, we find that *B. anthracis* Sterne kills *G. mellonella* in a dose-dependent manner (Figure 1A).

Our next step was to determine whether the survival rate of *G. mellonella* differed when injected with the wild-type *B. anthracis* Sterne or an attenuated mutant. We used the ΔpXO1 mutant as this strain lacks the pXO1 plasmid, which encodes the lethal and edema toxins, and is avirulent in mice (van Sorge et al., 2008; Levy et al., 2012). We injected a dose of ~5 × 10^4^ cfu per larva, which was mid-way between the two highest doses in our dose curve. This resulted in an overall survival rate around 20% when larvae were infected with wild-type *B. anthracis* Sterne (Figure 1B). In contrast, 70% of the larvae injected with the attenuated ΔpXO1 mutant survived, which was almost identical to the PBS-injected control larvae. We thus find that, consistent with the mammalian model, the ΔpXO1 mutant is severely attenuated for virulence in *G. mellonella*.

**Figure 2:**
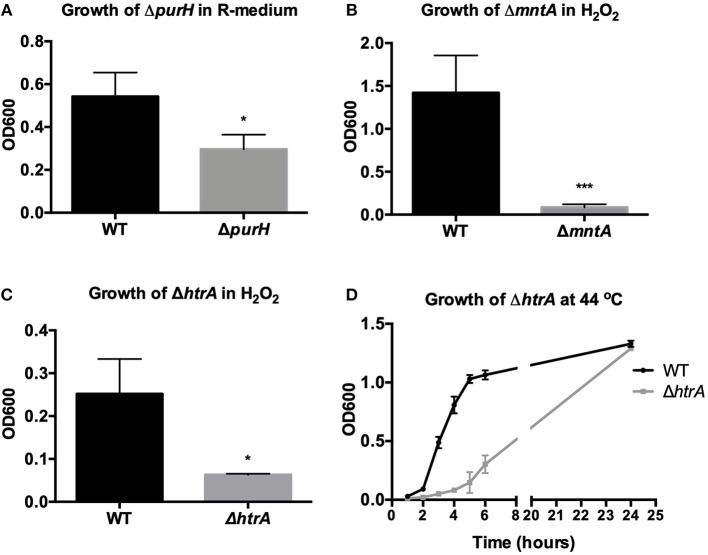
Validation of *in vitro* phenotype of reconstructed virulence mutants. Growth of wild-type *B. anthracis* Sterne (WT) was compared to the growth of **(A)** Δ*purH* in minimal R-medium after 24 h **(B)** Δ*mntA* in H_2_O_2_ after 24 h **(C)** Δ*htrA* in H_2_O_2_ after 8 h and **(D)** Δ*htrA* at 44°C in BHI. Assays were repeated three independent times for **(A,C,D)** and four independent times for **(B)** and are presented as mean ± SD. ^*^p < 0.05 and ^***^p < 0.001 by unpaired Student's *t*-test.

**Figure 3:**
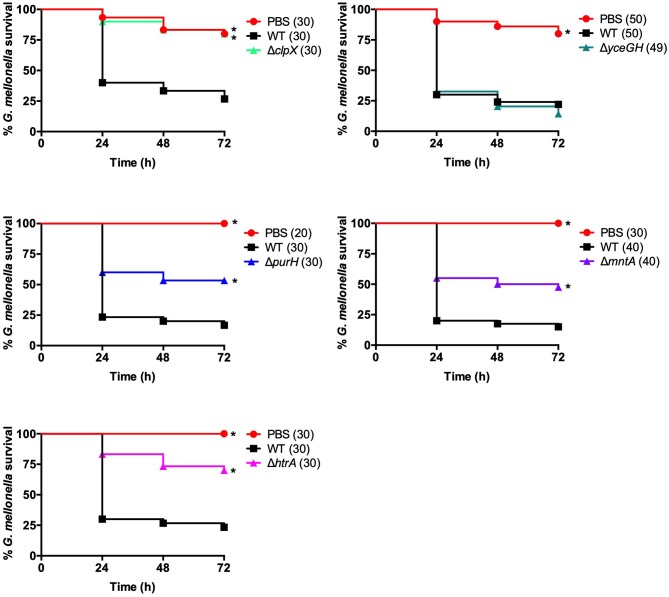
Mutants have attenuated virulence in *G. mellonella*. Survival of larvae injected with PBS, ~5 × 10^4^ cfu of wild-type *B. anthracis* Sterne (WT), or the indicated mutant. Each infection was repeated at least three independent times and total number of larvae for each condition are indicated in parentheses. ^*^Indicates statistically different survival than wild-type, *p* < 0.025 by the Mantel-Cox log rank test.

Although this was promising, we wanted to assess the ability of the *G. mellonella* model to discern changes in virulence beyond the ΔpXO1 mutant. The pXO1 plasmid encodes as many as 143 open reading frames, including the toxins, and it also plays a role in regulating chromosomal genes (Okinaka et al., 1999; Perego and Hoch, 2008). Therefore, its loss should have profound consequences on the virulence of *B. anthracis*. We wanted to assess whether less severe mutations would also demonstrate attenuated virulence in our *G. mellonella* infection model. We tested five mutant strains of *B. anthracis* Sterne with disruptions in chromosomal genes that had been previously implicated in virulence (Table 2). These chromosomal genes include *clpX*, an ATPase that functions as part of the ClpXP protease, and *yceGH*, which encodes the last two genes of a 6-gene cluster of tellurite resistance genes. Our lab has previously linked these genes to defense against AMPs (*clpX*) and ROS (*yceGH*) (McGillivray et al., 2009; Franks et al., 2014). We also looked at *purH*, which is critical for purine biosynthesis (Jenkins et al., 2011) along with *mntA*, part of a manganese transport system, and *htrA*, a serine protease, which are both important for defenses against ROS (Gat et al., 2005; Chitlaru et al., 2011). Four of these genes, *clpX, purH, mntA*, and *htrA*, had been constructed in a fully-virulent strain of *B. anthracis* (pXO1^+^, pXO2^+^) and shown to be attenuated in mammalian infection models so we were confident these represented true virulence determinants of *B. anthracis*. Mutations in *clpX* and *yceGH* already existed in our lab, and we reconstructed mutations in the other three genes in the Sterne strain. After disrupting *purH, mntA* and *htrA* using insertional mutagenesis, we confirmed that our reconstructed mutants had similar *in vitro* phenotypes as were previously published (Gat et al., 2005; Chitlaru et al., 2011; Jenkins et al., 2011). This included growth deficits in minimal R-medium for Δ*purH*, increased susceptibility to H_2_O_2_ for Δ*mntA* and Δ*htrA* and decreased growth at high temperature for Δ*htrA* (Figure 2). We injected each *G. mellonella* larva with ~5 × 10^4^ cfu of either the parental (wild-type) or mutant bacteria or a PBS control and found that all of the mutant strains with the exception of Δ*yceGH* exhibited decreased virulence relative to the wild-type strain (Figure 3).

**Table 2:** Chromosomal virulence mutants used in this study.

**Gene**	**Function**	**Phenotype of mutant**	***B. anthracis* strain used in mammlian infection models**	**Derivation of strain used in current study**
*clpX*	ATPase that functions as part of ClpXP protease	AMP susceptibility	Sterne (pX01^+^, pX02^−^) and Ames (pX01^+^, pX02^+^)	Constructed in McGillivray et al. (2009)
*yceGH*	Tellurium resistance	ROS susceptibility	Sterne (pX01^+^, pX02^−^)	Constructed in Franks et al. (2014)
*purH*	Purine biosynthesis	Nutritional deficits	Ames (pX01^+^, pX02^+^)	Reconstructed in this study
*mntA*	Transporter	ROS susceptibility	Vollum (pX01^+^, pX02^+^)	Reconstructed in this study
*htrA*	Protease	ROS susceptibility and growth deficits	Vollum (pX01^+^, pX02^+^)	Reconstructed in this study

### Competition Assay Using *G. mellonella*

**Figure 4:**
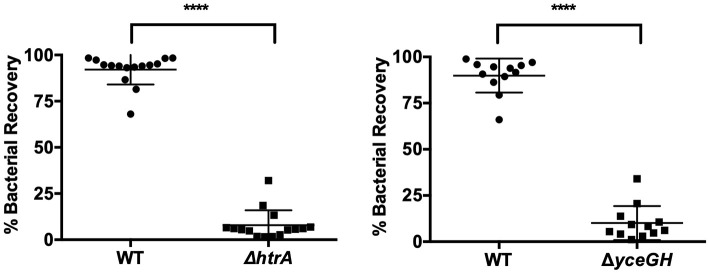
Attenuated virulence is seen in a competition model of infection. Relative survival of wild-type *B. anthracis* Sterne (WT) and either Δ*htrA* (left) or Δ*yceGH* (right) after being mixed at a 1:1 ratio and injected into each larva. Assays were repeated three independent times and all data were combined with each point representing an individual larva. ^****^p < 0.0001 by unpaired Student's *t*-test. Line represents mean ± SD.

In our earlier study with the Δ*yceGH* mutant, we saw decreased fitness in mice using a competition model of infection in which wild-type and the Δ*yceGH* strain were mixed at a 1:1 ratio and injected as a mixed culture into each mouse to determine relative survival of each bacterial strain (Franks et al., 2014). To develop a comparable competition model in *G. mellonella*, we mixed wild-type and mutant *B. anthracis* Sterne (either Δ*htrA* or Δ*yceGH*) at a 1:1 ratio. Ten microliters of the mixed culture (~1 × 10^5^ total cfu) were injected into each larva and the larvae were then incubated for 6 h at 37°C. We chose 6 h as this was long enough for the infection to become established but short enough to preclude any larval mortality. Larvae were then rinsed in 70% ethanol followed by sterile water to help minimize contamination by surface bacteria before being homogenized in PBS via bead beating. Homogenates were plated on BHI and BHI-antibiotic plates (Erm5 for Δ*htrA* and Kan50 for Δ*yceGH*) and the cfu recovered for each strain was calculated. Both the Δ*htrA* and Δ*yceGH* mutants showed decreased fitness in this model with only about 10% of the recovered cfu belonging to the mutant strains (Figure 4). As a control to make sure that homogenization did not impact relative bacterial survival, we bead-beated and plated a portion of the initial mixed culture prior to injection in the larva and saw no difference in relative survival between the wild-type and mutant strains (data not shown). We conclude that a competition model is also a viable infection model for *G. mellonella* and may be more sensitive in discerning relative differences in strain fitness than the survival assay.

### Use of *G. mellonella* to Assess Virulence

**Figure 5:**
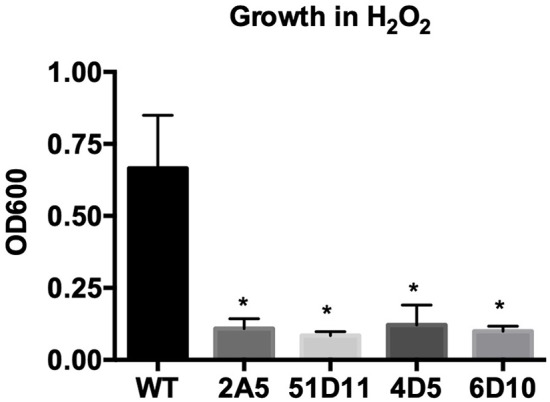
Transposon mutants have increased susceptibility to H_2_O_2_. Wild-type *B. anthracis* Sterne (WT) and transposon mutants were grown in 0.01% H_2_O_2_. Assays were repeated four independent times and data are presented as mean ± SD. ^*^p < 0.05, from WT by one-way ANOVA followed by Dunnett's *post-hoc* analysis.

**Figure 6:**
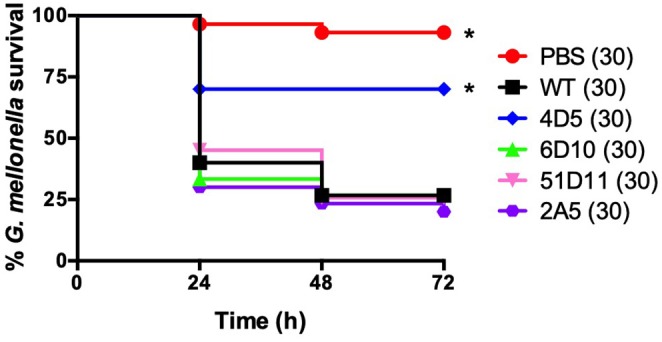
The 4D5 transposon mutant has decreased virulence in *G. mellonella*. Survival of larvae injected with PBS or ~5 × 10^4^ cfu of wild-type *B. anthracis* Sterne (WT) or the indicated transposon mutant. Each infection was repeated at least three independent times and total number of larvae for each condition are indicated in parentheses. ^*^Indicates statistically different survival than wild-type, *p* < 0.01 by the Mantel-Cox log rank test.

Our next goal was to determine whether *G. mellonella* could be used to assess virulence in transposon mutants that displayed promising *in vitro* phenotypes. Previously, we had conducted several *in vitro* screens using a library of 5,000 transposon mutants. These screens had yielded a total of 44 mutants that either had altered hemolytic activity, were less virulent in the nematode *Caenorhabditis elegans*, or had decreased proteolytic activity as evidenced by their inability to degrade casein in 5% milk plates (McGillivray et al., 2009; Franks et al., 2014); and unpublished data). Two transposon mutants pulled from these screens ultimately led to our characterization of the role of *clpX* (McGillivray et al., 2009) and *yceGH* in virulence (Franks et al., 2014), but this left 42 mutants with potentially interesting phenotypes to be investigated. In order to narrow this number down further, we screened these 42 mutants for susceptibility to hydrogen peroxide. H_2_O_2_ is one of the many ROS that are produced by the innate immune system and serve as a critical defense against bacterial pathogens (Lambeth, 2004). Four of these transposon mutants, 2A5, 51D11, 4D5, and 6D10, were consistently attenuated in their ability to grow in the presence H_2_O_2_ (Figure 5), although there was no difference in growth in plain BHI (data not shown). We next tested these 4 mutants in our *G. mellonella* survival model. Only one of these mutants, 4D5, was attenuated for virulence relative to the wild-type strain (Figure 6). We determined the site of transposon insertion for 4D5 and found it disrupted the *asbC* gene of the petrobactin biosynthesis operon *asbABCDEF*. Notably, loss of this operon has been previously linked to both reduced virulence in mice (Cendrowski et al., 2004) and diminished defense against oxidative stress (Hagan et al., 2018). Therefore, we conclude that *G. mellonella* can effectively identify attenuated virulence mutants of *B. anthracis* Sterne.

## Discussion

A major goal in our development of a *G. mellonella* infection model is to use it to identify novel virulence genes in *B. anthracis* Sterne. This in turn could help identify promising drug targets as part of an antimicrobial strategy aimed at disarming pathogens (Clatworthy et al., 2007). A common strategy for the identification of novel virulence factors is to screen a library of several thousand genetic mutants that cover the entire bacterial genome. *In vitro* screens are relatively easy to carry out in a high throughput manner, but if too many hits are returned it can be challenging to decide which ones to further characterize, particularly when the mutations are in hypothetical proteins or genes of unknown function. The ability to first conduct an *in vitro* screen and then pass any hits through a practical *in vivo* infection model would help prioritize mutants for follow-up studies. We have applied this strategy by screening a small pool of transposon mutants first *in vitro* using H_2_O_2_ and then *in vivo* in *G. mellonella*. One of these mutants, 4D5, showed attenuation in both screens. This mutant had a disruption of the petrobactin biosynthetic operon *asbABCDEF*, which has been previously linked to both resistance to oxidative stress (Hagan et al., 2018) and virulence in a mouse model of infection (Cendrowski et al., 2004). While this is not a novel virulence determinant, it confirms that this strategy can effectively identify a virulence mutant of *B. anthracis* in an unbiased manner.

We developed two different *G. mellonella* infection models to assess virulence. Both the survival and competition assays were effective as each discerned attenuated virulence in *B. anthracis* Sterne using known virulence mutants. Of the two, the competition assay is likely more sensitive in detecting changes in bacterial fitness, as we were able to see decreased virulence of the Δ*yceGH* mutant using this assay but not with the survival assay. A recent study established a health index scoring system for *G. mellonella* that, in addition to survival, assesses activity, cocoon formation and melanization; using these additional metrics may increase the sensitivity of the assay rather than relying on survival as the sole data point (Loh et al., 2013). Even so, we chose to use the survival assay when assessing the virulence of our transposon mutants in order to focus on mutants with a strong phenotype.

We obtained *G. mellonella* larvae from an online bait shop. This was easy and cost-effective, but allowed no control over the age, rearing conditions or genetic background of the *G. mellonella* we received. This could lead to variability from assay to assay even when using a standardized larval weight. In the *G. mellonella* survival assays we performed for this study, injection of ~5 × 10^4^ cfu of wild-type *B. anthracis* Sterne resulted in a median larval survival rate of 26.3% with a standard deviation of 14.5% for a total of 160 worms over 16 independent experiments. Therefore, despite some variability in survival rates, the assay was consistent enough to be reliable. In order to make the larvae easier to inject, we stored them at 4°C to induce a torpid state prior to handling. An earlier study found that exposure of *G. mellonella* larvae to temperatures of 4°C before infection with *Candida albicans* resulted in increased larval survival, which may have been mediated by increased expression of AMPs and increased hemocyte density at the lower temperature (Mowlds and Kavanagh, 2008). Thus, it is possible the exposure to decreased temperature could have affected the larval response to infection. Regardless, since all of our larvae were treated in the same manner, our results should remain comparatively valid. We will note that even when the larvae were immobile, we still had some injection-induced trauma. This is can be seen when comparing the 98% survival rate in our uninjected controls across all of our assays (100 larvae total) to the 87% survival rate in our PBS-injected control groups (180 larvae total). However, a survival rate of <80% in the PBS-injected group was rare, occurring in only two of our assays, and is likely indicative of poor injection technique or underlying health issues in the larvae and should be taken into consideration, especially if it is a recurring issue.

There are a number of advantages to using an invertebrate infection model including reduced infrastructure needs and fewer regulatory requirements. We find that *G. mellonella* is a promising animal model for *B. anthracis* Sterne with results that correlate well with those in mammalian models of infection. We also see a number of advantages with the *G. mellonella* model. The larvae are large enough to be directly injected allowing for precise pathogen exposure and more consistent results. *G. mellonella* can also be incubated at 37°C, which is important for temperature-sensitive gene expression in bacterial pathogens (Shapiro and Cowen, 2012). However, there are also some drawbacks. Unlike *C. elegans, G. mellonella* does not offer an ingestion route of infection, as a prior attempt to orally infect *G. mellonella* with *B. anthracis* Sterne proved unsuccessful (Fedhila et al., 2010). There are also limited genetic tools in *G. mellonella* making it difficult to manipulate the host side of the infection model, in contrast to *C. elegans* where RNA-mediated interference is a well-established technique for knocking down host gene expression (Glavis-Bloom et al., 2012). Therefore, we see *G. mellonella* as a useful complement to the other established infection models, both vertebrate and invertebrate, that currently exist for *B. anthracis*. Ultimately, we hope the development of the *G. mellonella* infection model will allow us to expand our understanding of *B. anthracis* virulence mechanisms while decreasing our reliance on mammalian models of infection.

## Data Availability Statement

The raw data underlying all figures presented in this publication can be found at https://doi.org/10.6084/m9.figshare.c.4670009.

## Author Contributions

SM conceived of the study, designed the experiments, and wrote the manuscript. JM and MR performed the experiments and also contributed to data analysis, experimental design, and manuscript editing.

### Conflict of Interest

The authors declare that the research was conducted in the absence of any commercial or financial relationships that could be construed as a potential conflict of interest.
